# Regularity and Predictability of Human Mobility in Personal Space

**DOI:** 10.1371/journal.pone.0090256

**Published:** 2014-02-27

**Authors:** Daniel Austin, Robin M. Cross, Tamara Hayes, Jeffrey Kaye

**Affiliations:** 1 Oregon Health and Science University, Department of Biomedical Engineering, Portland, Oregon, United States of America; 2 Oregon State University, Agricultural and Resource Economics, Corvallis, Oregon, United States of America; 3 Oregon Health and Science University, Department of Neurology, Portland, Oregon, United States of America; National Research & Technology Council, Argentina

## Abstract

Fundamental laws governing human mobility have many important applications such as forecasting and controlling epidemics or optimizing transportation systems. These mobility patterns, studied in the context of out of home activity during travel or social interactions with observations recorded from cell phone use or diffusion of money, suggest that in extra-personal space humans follow a high degree of temporal and spatial regularity – most often in the form of time-independent universal scaling laws. Here we show that mobility patterns of older individuals in their home also show a high degree of predictability and regularity, although in a different way than has been reported for out-of-home mobility. Studying a data set of almost 15 million observations from 19 adults spanning up to 5 years of unobtrusive longitudinal home activity monitoring, we find that in-home mobility is not well represented by a universal scaling law, but that significant structure (predictability and regularity) is uncovered when explicitly accounting for contextual data in a model of in-home mobility. These results suggest that human mobility in personal space is highly stereotyped, and that monitoring discontinuities in routine room-level mobility patterns may provide an opportunity to predict individual human health and functional status or detect adverse events and trends.

## Introduction

Many factors influence human mobility, spanning the continuum from regular and predictable commitments (e.g., commuting for work or taking a child to school) to unforeseen circumstances (e.g., travelling to help a sick relative or pausing to fix a flat tire) while also encapsulating individuals' preferences, wants, needs, and contextual effects (e.g., weather conditions or current health status). Despite the seemingly diverse array of reasons for which individuals move around[1], [2], [3], [4], [5], [6], a large body of work has found significant regularity and predictability in human mobility patterns, primarily in the form of scaling properties and power laws[2], [3], [5], [7], [8], [9], [10], [11] using location data collected predominantly from cell phones[7], [12], [13], [14]. These findings have importance to a diverse array of applications such as optimization of transportation systems[15], [16] and controlling the spread of infectious disease[17], [18], [19]. To date, these studies have focused on human mobility outside of the personal home space. In part, this reflects the commonly used proxies for human mobility, such as cell phone records, which lack the spatial and temporal resolution to resolve movements on the scale present in home space. However, much of the population spends a significant proportion of their time at home – especially as they age[20] – suggesting that mobility in the home is an important facet of human behavior.

Recent advances in ubiquitous computing and in-home monitoring have provided opportunities to monitor individuals in their personal home space both passively and unobtrusively via motion sensors and other devices[21], [22], [23], [24] providing the opportunity to study continuous behavioral characteristics in the home setting for the first time (see Fig. S1 for an example of an in-home monitoring platform floor plan and setup). Current methods for in-home monitoring are somewhat different than out-of-home monitoring in that they lack a common measurement system for all individuals, such as common cell phone towers used to measure mobility out-of-home[7], [8], [12], [13], [14]. This is due in part to heterogeneity of the home space (size, furniture placement, etc.) and in-home sensor networks (number of sensors deployed, sensor placement in the home, etc.), making the spatial aspect of mobility less comparable across individuals. In particular, we can readily account for (as in the contextual model; see below) the opportunity space an individual has in which to move temporally (e.g., size, number of sensors, average recorded mobility), but it is difficult to make meaningful cross-sectional comparisons when individuals' spatial locations do not map across people (e.g., not everyone has a computer room or second bedroom). For this reason we focused our investigation on the temporal regularity and predictability of mobility, *m_i_*, where mobility is defined as the number of times an individual moves between different rooms in their home - a count variable quantifying an individual's number of movements in a specified time interval *i*.

We focused our investigation on two main questions, both of which address aspects of predictability and regularity in human mobility not previously examined. First, we sought to determine whether scaling laws in human mobility similar to those demonstrated outside the home also hold inside the home. Second, we investigated whether including context in a model of human mobility uncovered regularity not accounted for by modeling mobility with a single, time independent power law mobility distribution. We find that while a power law is not a plausible representation for the observed in-home mobility data, by explicitly including context in a model of human mobility we obtain a high level of predictability and uncover structural regularity not previously reported. These results suggest that in-home mobility is also highly stereotyped, albeit in a different way, which may have applications to predicting individual human health and functional status[25], [26] by detecting adverse events or trends[7] and in conducting more meaningful clinical trials[27], [28].

## Results and Discussion

For both investigations, we used a dataset consisting of 14,920,560 measurements of mobility recorded in two-minute intervals from 19 older adults monitored for up to 5 years in their own homes. Data were gathered from participants in the Intelligent Systems for Assessing Aging Changes (ISAAC) study[20], a longitudinal cohort study of naturalistic aging using unobtrusive embedded home activity sensing (see Methods and Supporting Information S1 for further details about the data, study, and study participants).

### Power Law

**Figure 1:**
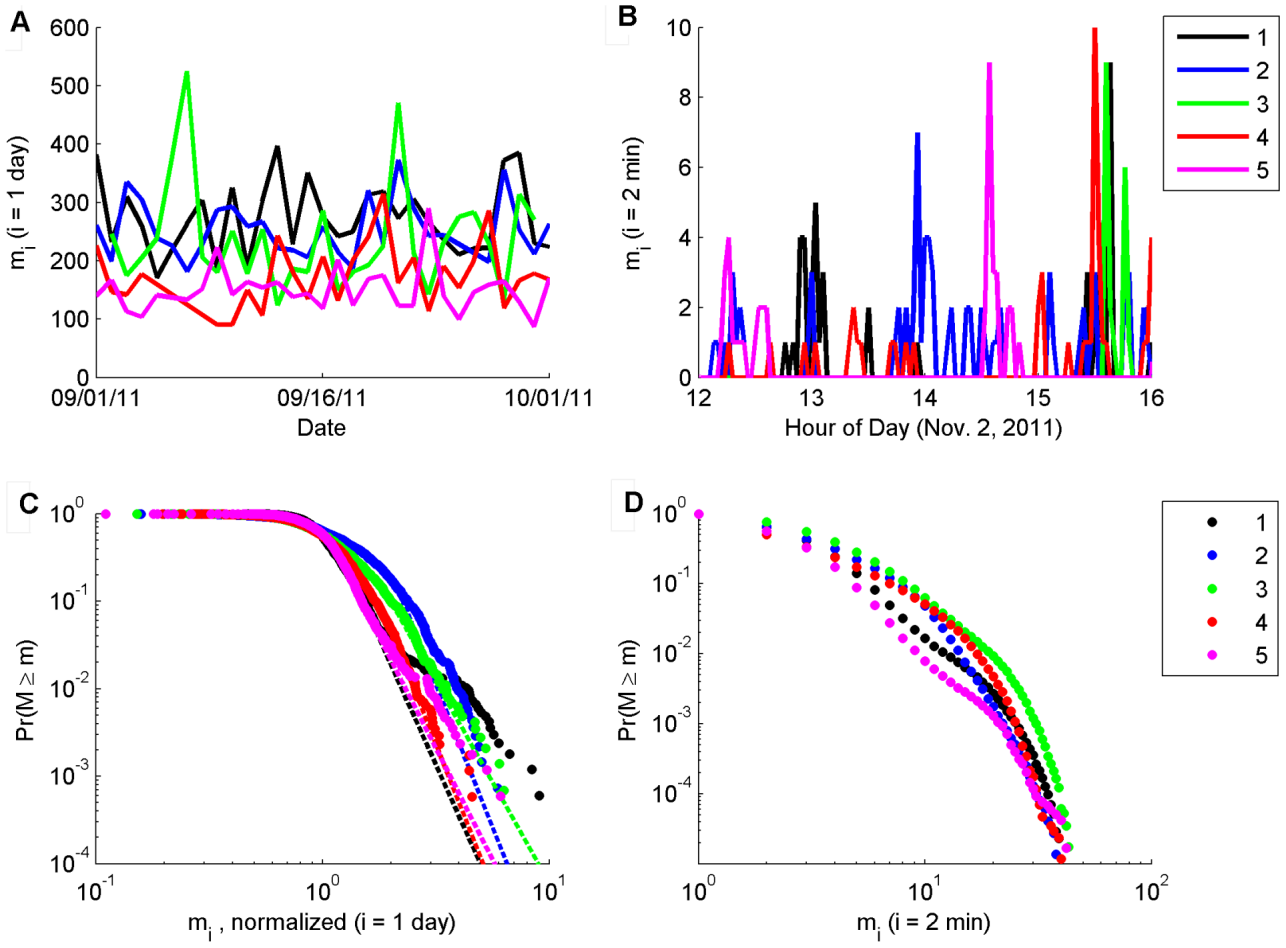
A snapshot of individual in-home mobility patterns. Time series plots of in-home mobility for 5 different participants (color coded) for daily-mobility (A) over 31 days starting November 1st of 2011 (top left) and (B) 2-minute increments for four hours of November 2, 2011. Mobility, in units of room-transitions, at the day level is impulsive occasionally changing by a factor of 2 or more. Mobility at the 2-minute level is also impulsive and demonstrates that day-level mobility is comprised of periods of little-to-no mobility interspersed with bursts of movement. (C) Day-level data for 5 representative homes is shown (circles) with best fit power laws (dashed lines), indicating good fit for individual homes but not across homes. Note that as we have plotted the cumulative distribution function for the power laws, the slopes in the plot are −α+1 for each participant's value of α. (D) Data at the two-minute level was not consistent with a power law distribution but is still heavy-tailed. Figs. S2–S4 show similar plots for the 14 homes not show here.

The mobility data over time, *m_i_*, for sampling intervals of one-day and two-minutes are shown in Fig. 1, where the sampling intervals were chosen to exemplify both gross (one-day) and fine (two-minute) grained mobility patterns. Mobility over the course of the day is comprised of bursts of movement separated by periods of little or no movement, suggesting that the large swings in day level mobility are driven by the number and size of mobility “bursts” at the two-minute level. The episodic nature of the mobility patterns at both sampling intervals coupled with results demonstrating power law behavior for out-of-home mobility[8], [9] suggested that a double truncated power law, P(*m)*∼*m^−α^* for *m_min_*≤*m*≤*m_max_*, could be a reasonable characterization of the data. The double truncation is suggested on the upper side by a physiological maximum speed of an individual, which limits the amount of possible mobility in a fixed interval, and on the lower side since most empirical data tend to follow a power law only in the tail of the distribution[29]. We found that a power law was a reasonable fit for 17 of the 19 *individuals*' mobility measured at the day level (the sum of two minute mobility samples over 24 hours; shown in Fig. 1 for five homes and Figs. S2–S4 for the remaining 14 homes) when normalizing the data by the individual specific median mobility and number of sensors in the home (see Supporting Information S1 for discussion of the normalization). However, in contrast to results reported for out-of-home mobility[8], the mobility *across* subjects did not collapse into a single power law distribution after normalization. Instead, significant differences were found in all three parameters of the distributions (*m_min_*, *m_max_*, and *α*; see Supporting Information S1). This suggests no universal scaling exponent governs human mobility in the home (see Supporting Information S1 for further discussion), but indicates that a high degree of individual regularity still exists.

A power law was inconsistent with all mobility data when sampled at two-minute intervals, demonstrating that the impulsive nature of in-home mobility on fine-grained time scales (Fig. 1) is not well approximated by a single, individual-specific, and time-independent power law density. This finding was somewhat surprising in light of the scaling laws found in many prior studies on human mobility or behavior in general (e.g., [1], [8], [9]), and further comments are in order. For the day-level power law analysis, there were 1090 samples (days of data) per home on average used to fit the power laws. For the 2-minute analyses, there were 785,292 samples - of which 67,535 were nonzero - on average, per home. The number of samples is an important concern because the standard test for whether a power law is a plausible fit for data is based on assuming a power law holds as the null hypothesis[8], [29] and only rejecting this null hypothesis if there is enough evidence in the data to do so. As a result, it is much easier, statistically speaking, to not reject the null with a small number of samples. Because of this, we believe the results presented at the 2-minute level of “no plausible power law” are more accurate than those presented at the day level since we had so many more samples to potentially reject the null hypothesis at the 2-minute level. This may point to a fundamental difference in the phenomena of in-home and out-of-home mobility, but further work is needed to investigate this (see Supporting Information S1 for further discussion).

### Contextual Model

In light of the lack of evidence for a universal scaling law for in-home mobility, we investigated the hypothesis that explicitly modeling the relationship between activity context and mobility would uncover regular and predictable structure in human mobility in-home. As a power-law density was not a good model for mobility and because *m_i_* is a non-negative integer valued variable, we used a negative binomial regression model[30] (see Supporting Information S1 for alternative models), where the probability of *m_i_* – the observed mobility in a two-minute interval at sample *i* – follows a negative binomial distribution:
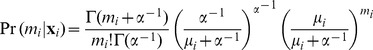
(1)where ***x*** is a vector of explanatory variables, μ is the expected value of the mobility distribution satisfying 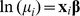, 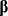 are the model parameters describing the individual contributions of each explanatory variable, *α* is a dispersion parameter controlling the conditional variance of the mobility distribution, Var(*m_i_|* ***x*** *_i_*) = *μ_i_+αμ_i_^2^*, Γ is the gamma function, and *i* indexes the observations. Eighty-eight explanatory variables (see Supporting Information S1) which we hypothesized could drive human mobility and be reliably measured were selected, representing seven general categories: behavioral (e.g., walking speed), weather (e.g., temperature, precipitation), self-report (e.g., age, health status), peer-reference (e.g., walking speed of the peer group), time-dependence (e.g., time, lagged variables), missing data, and physical environment (e.g., home size). Variables from the first five categories were included to directly account for the influence between observed context, observed phenomena, and mobility, while the last two categories were included to account for missing data and known heterogeneity across both subjects and home space. While eighty-eight variables may appear too large a number to include without the risk of over fitting the model to the data, the large number of data points available with which to estimate the parameters prevents this (see also Supporting Information S1 for further discussion of this important consideration).

The contextual model allows the determination of two important questions: 1) is in-home mobility predictable and 2) is in-home mobility regular? If the model uncovers predictability and regularity in human mobility, then we can infer that context is an important part of human mobility patterns. Further, a context-based model that adequately approximates human mobility allows inferences on the relationship between contextual variables and mobility. To investigate these questions, we fit the data to the model described by equation (1) and found the model to be both statistically significant and an accurate representation of the data (see Supporting Information S1).

**Figure 2:**
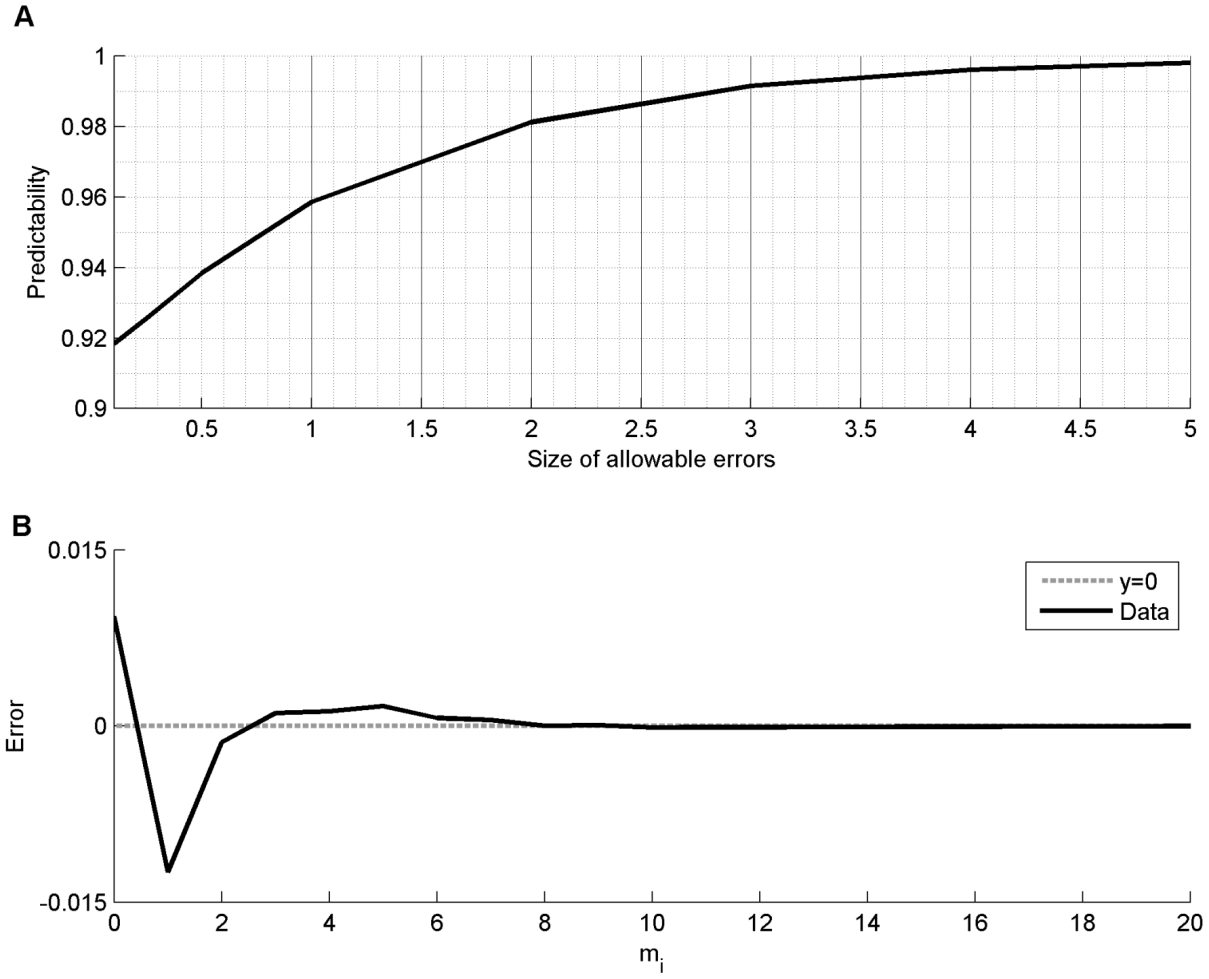
The predictability of in-home mobility. (a) Predictability of in-home mobility, defined as the proportion of correct estimates, is shown as a function of allowable error. For example, 95.8% of mobility estimates are within 1 of observed values, and over 99% of mobility estimates are within 3 of observed values. (b) The difference between estimated and observed probabilities are shown (black line) as a function of mobility. The *y = 0* line (gray dashed line) represents perfect predictability. The regression model over estimates 0's by 1%, underestimates 1's by 1.2%, and is off by less than 0.2% for the rest of the mobility values. Confidence intervals on the predictability have been omitted as they are too small to distinguish from the figure traces (see Tables S3–S4).

Predictability can be defined in many ways, perhaps most often with an entropy-based definition. This method has the advantage of not requiring an explicit construction of a model that can make predictions in order to determine the theoretical predictability of a time series[3]. However, the contextual model can predict mobility on a sample-by-sample basis, allowing predictability to be assessed directly as the accuracy of the model in making predictions. We evaluate predictability using two different but complementary measures of model accuracy (see Supporting Information S1 for full details). The first measure of predictability directly assesses prediction accuracy in the contextual model. Specifically, we define the first measure of predictability as the proportion of correctly predicted mobility samples. This measure is best represented as a function of the size of allowable prediction error (e.g., the size of the prediction error allowed for a prediction to be considered correct; Fig 2A). In our data set, 91.4% of the observations are 0 (no mobility) with a range of observed mobility values from 0 to 41 across the entire data set. Therefore, predictability must be substantially higher than 91.4% to be meaningful. We found that if the allowed prediction error is 3 transitions or less (a 7% error with respect to the range of data), then the model has over 99% predictability (Fig. 2A; see also Supporting Information S1). We also quantified predictability as the difference in model predicted mobility counts and observed mobility counts in our data set (Fig. 2B). We found that the model overestimates periods of no movement by 1%, underestimates periods with a single movement by 1.2%, and is within 0.2% or less of observed values for all other values of mobility. Taken together, these results demonstrate a high degree of predictability is present in human home-space mobility when context is taken into account.

Regularity can be quantified in several ways, perhaps most often related to the return probability or time spent in a highly frequented location[3], [8], [31], [32], a form of spatio-temporal regularity. As we have not explicitly taken location into account in our analyses, we instead investigate temporal regularity as measured by the presence of rhythms or periodicities[33], [34], [35] in mobility that may be biologically or contextually determined, and the existence of correlations on multiple time scales. The inclusion of time variables (e.g., hour of day) and lagged activity variables at different time scales (e.g., a day ago or a week ago) in the contextual model allows for the determination of this type of regularity in human mobility. In particular, we found that all of the behavioral variables exhibited some effect on mobility at different time lags spanning a time-dependence of as little as two-minutes (one sample) to one month (the longest time lag included in the model) suggesting that the mobility exhibits a form of autocorrelation on multiple time scales. We also found that time influenced mobility on different scales with hour of the day, month, and year all impacting the amount of observed mobility (see Supporting Information S1 for a full description of the effects of different variables). Combining these insights gives strong evidence for regularity in mobility patterns as exhibited both by circadian, infradian, and ultradian cycles and with correlations on multiple time scales. In particular, this suggests that in the absence of extenuating circumstances (characterized as the other variables used in the contextual model) humans tend to have highly regular mobility patterns (e.g., moving less at night, more in the morning, and so on).

**Figure 3:**
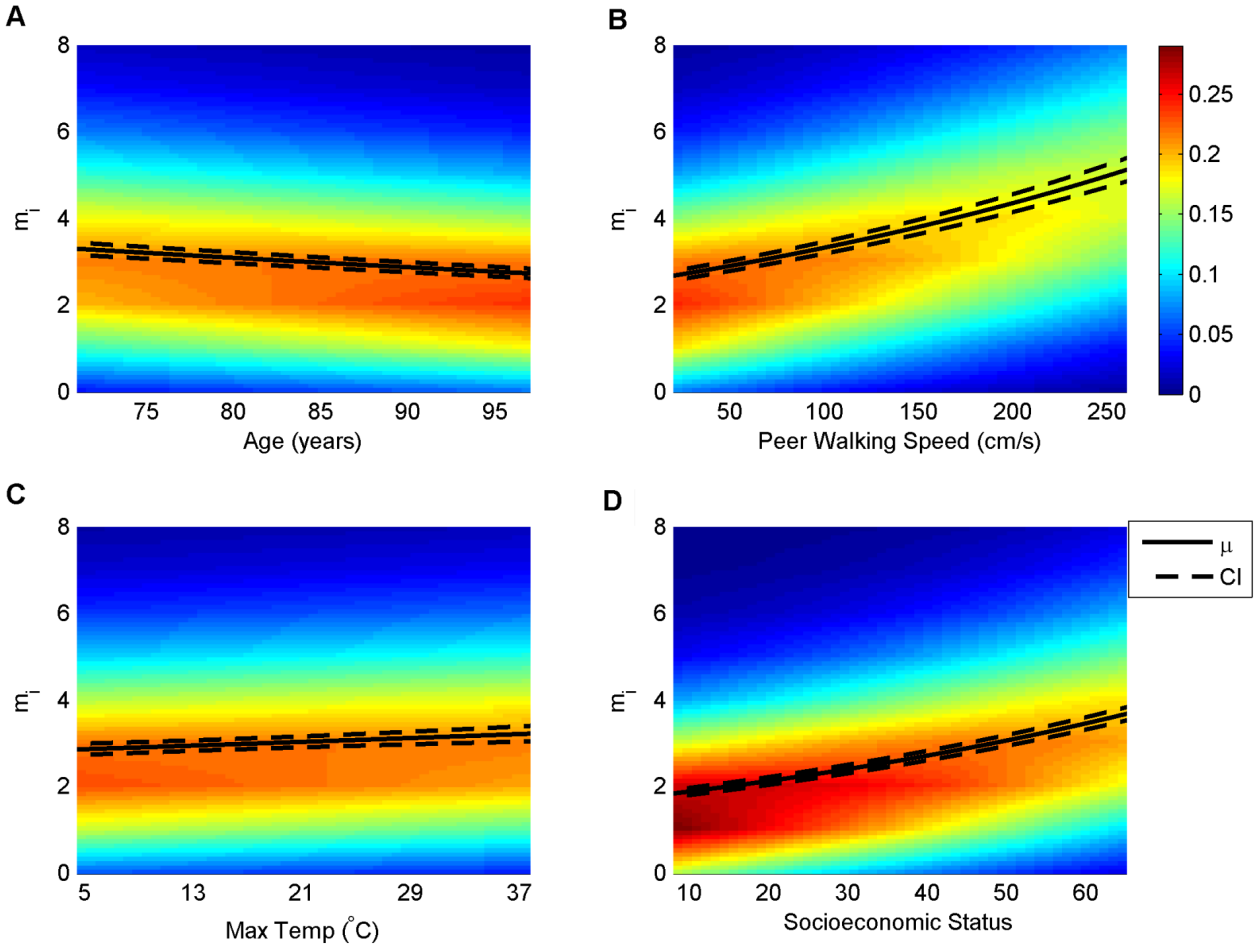
The effect of life context. Probability density (color represents density; discrete probabilities were linearly interpolated for graphical clarity) of mobility (y-axes) as a function of four different contextual variables (x-axes) that vary across different time scales, holding all other variables at constant values (see Supporting Information S1 for details). The mean function, μ (black trace) with 95% confidence intervals (CI; dashed black trace), has been overlaid on the density to show central tendency in each panel. (A) Mobility declines with increasing age, where expected mobility decreases by a factor of 0.83 as age increases from 71 to 97 years. (B) Mobility increases with increasing walking speed among the peer reference group (expected mobility increases by a factor of 1.9 as peer referenced walking speed increases from 20 cm/s to 260 cm/s), (C) maximum daily outdoor temperature (expected mobility increases by a factor of 1.12 as maximum temperature increases from 5°C to 37°C), and (D) socioeconomic status (expected mobility doubles as socioeconomic status increases from a score of 8 to 65).

**Figure 4:**
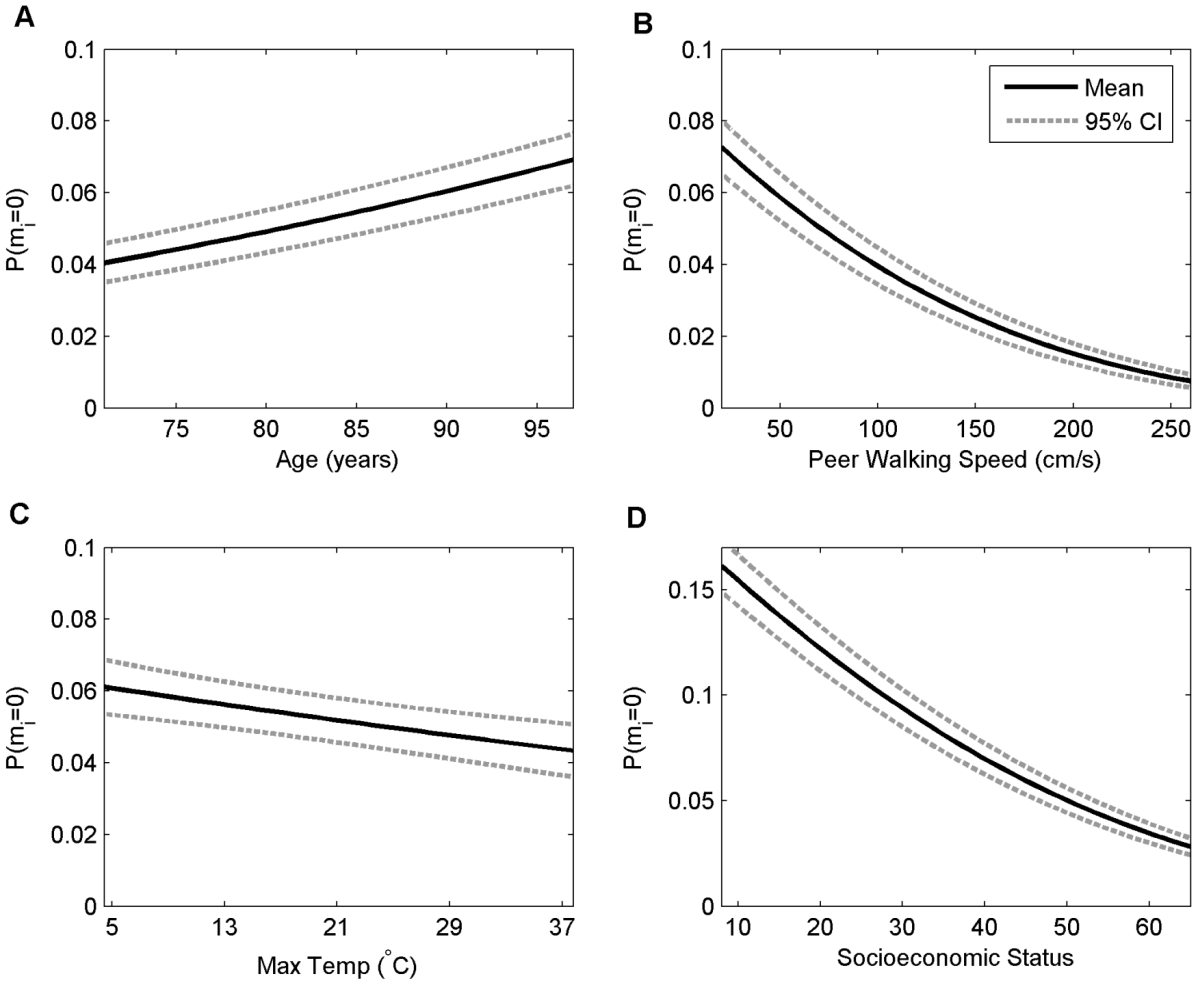
The probability of not moving in-home. The estimated probability of no movement with 95% confidence intervals (CI) as a function of 4 different contextual variables (x-axes), holding all other variables at constant values (including gender  =  female, same as values in Fig. 3, see Supporting Information S1 for details). (A) The probability of no movement increases by a factor of 1.7 as age increases from 71 to 97 years. (B) The probability of not moving decreases with increasing walking speed of the peer reference group (probability decreases by a factor of 0.1 as peer referenced walking speed increases from 20 cm/s to 260 cm/s), (C) increasing maximum daily outdoor temperature (probability decreases by a factor of 0.71 as maximum outdoor temperature increases from 5°C to 37°C), and (D) increasing socioeconomic status (probability decreases by a factor of 0.17 as socioeconomic status increases from 8 to 65). Socioeconomic status has been plotted with a different y axis range than the other three variables as it has a larger range.

Combining the evidence for the high degree of regularity and predictability in human mobility in-home supports the hypothesis that context is an important driving force behind when and how much humans move in their homes. One additional advantage of the regression framework is that the model parameters estimated from the data allow inferences about how mobility changes with changing context. Fig. 3 shows how the mobility density changes for changing values of context for four different contextual variables that change on different time scales (while all other variables are held at specified values; see Supporting Information S1). The time scales of the different contextual variables are as follows: Age linearly increases with time for all participants, maximum temperature can vary day-to-day, peer-walking speed can change from two-minute sample to two-minute sample, and socioeconomic status is relatively fixed at this point in participants' lives. Increasing age is associated with decreasing mobility, whereas increasing temperature, peer walking speed, and socioeconomic status are all associated with increased mobility (Fig. 3). Another important question for which the model can make a prediction is: what is the effect of context on the probability of not moving? Fig. 4 shows the probability of not moving for changing context using the same data as shown in Fig. 3. Not surprisingly, as expected mobility increases as the probability of not moving decreases.

Our results suggest that while in-home mobility does not appear to follow a universal scaling law, accounting for context uncovers both regularity and predictability in this mobility in a way in which a single time-independent scaling distribution cannot. Further, in-home mobility is highly stereotyped both within and across subjects when context is taken into account. This result is potentially useful for behavioral forecasting (predicting patterns of mobility over time), especially since deviations from highly stereotyped in-home behavior may have significant and broad application in predicting both acute and long-term illness or wellness such as in predicting personal health status[36], [37] or in conducting more meaningful clinical trials[27], [28].

## Methods

All study participants whose data we used in this study were enrolled in the Intelligent Systems for Assessing Aging Changes (ISAAC) study, a longitudinal cohort study of naturalistic aging described in detail elsewhere[20]. All participants provided written informed consent and the ISAAC study was approved by the Oregon Health & Science University Institutional Review Board (IRB# 2353). A more detailed description of the ISAAC study and participant pool along with a detailed description of the data analysis used in this study not already described in the text is included in the Supporting Information S1.

## Supporting Information

Figure S1 **An instrumented home space.** Example floor plan showing location of motion sensors (red symbols), walking speed sensors (teal symbols), and contact sensors (purple rectangles) with associated area (text) and approximate center of sensor field of view (cross hair symbol). The computer symbol and router (purple) icons represent the placement of the data computer, router, and transceiver.
(TIF)

Figure S2 **Another snapshot of individual in-home mobility patterns.** Time series plots of in-home mobility for participants 6–10 (color coded) for (**A**) daily-mobility over 31 days starting November 1st of 2011 and (**B**) 2-minute increments for four hours of November 2, 2011. (**C**) Day-level data for 5 homes is shown (circles) with best fit power laws (dashed lines), indicating good fit for individual homes but not across homes. Note that as we have plotted the cumulative distribution function for the power laws, the slopes in the plot are −α+1 for each participant's value of α. (**D**) Data at the two-minute level was not consistent with a power law distribution but is still heavy-tailed. Participant 6's data was not consistent with a power law at the day level (black) with no power law trace in (**C**). Dates shown were chosen to show one month worth of data for all homes.
(TIF)

Figure S3 **A third snapshot of individual in-home mobility patterns.** Time series plots of in-home mobility for participants 11–14 (color coded) for (**A**) daily-mobility over 31 days starting July 7, 2009 and (**B**) 2-minute increments for four hours of July 7, 2009. (**C**) Day-level data for 4 homes is shown (circles) with best fit power laws (dashed lines), indicating good fit for individual homes but not across homes. Note that as we have plotted the cumulative distribution function for the power laws, the slopes in the plot are −α+1 for each participant's value of α. (**D**) Data at the two-minute level was not consistent with a power law distribution but is still heavy-tailed Dates shown were chosen to show one month worth of data for all homes.
(TIF)

Figure S4 **A fourth snapshot of individual in-home mobility patterns.** Time series plots of in-home mobility for participants 15–19 (color coded) for (**A**) daily-mobility over 31 days starting August 1, 2009 and (**B**) 2-minute increments for August 1, 2009. (C) Day-level data for 5 homes is shown (circles) with best fit power laws (dashed lines), indicating good fit for individual homes but not across homes. Note that as we have plotted the cumulative distribution function for the power laws, the slopes in the plot are −α+1 for each participant's value of α. (D) Data at the two-minute level was not consistent with a power law distribution but is still heavy-tailed Participant 18's data was not consistent with a power law at the day level (red) with no power law trace in (**c**). Dates shown were chosen to show one month worth of data for all homes.
(TIF)

Table S1 **Power law results for mobility in one day increments after normalization for home-specific median mobility and number of sensors installed in the home.** The distributional parameters: *α*, *m_min_*, and *m_max_* are reported along with standard errors *α_SE_* and *m_min,SE_* and the *p* value for the fit (higher *p* values suggest a better fit). The largest observed value in the data for each home, *max*(*m*) is reported for comparison with the largest value for which a power law is consistent (*m_max_*) to quantify the range over which a power law holds. The standard errors combined with the parameter estimates show that even after normalizing for participant and measurement specific effects, there is not a universal power law. Homes 6 and 18 were not consistent with a power law.
(DOC)

Table S2 **Summary of model.** Parameters (mean and 95% confidence intervals), significance at the 5% level (denoted by *), interpretation as a percentage change in the expected mobility due to a *unit* change in the associated independent variable with all other variables held constant, units, and description of variables.
(DOC)

Table S3 **Proportion of correct mobility estimates with 95% confidence intervals (CI) for different values of largest allowable estimation error (residual size; see** **Fig. 2A** **).**
(DOC)

Table S4 **Estimated, observed, and difference in mobility probabilities according to mobility value (see** **Fig. 2B** **).**
(DOC)

Supporting Information S1 **Additional information on data, methods, and results.**
(DOC)
