# Supplementary figures and images for: Regularity and Predictability of Human Mobility in Personal Space
Source: PLoS One. 2014 Feb 27;9(2):e90256. doi: 10.1371/journal.pone.0090256 (PMC3937357; doi:10.1371/journal.pone.0090256)

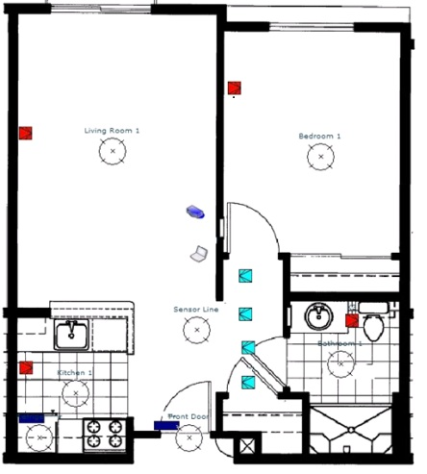

Supplement: Figure S1 — An instrumented home space. Example floor plan showing location of motion sensors (red symbols), walking speed sensors (teal symbols), and contact sensors (purple rectangles) with associated area (text) and approximate center of sensor field of view (cross hair symbol). The computer symbol and router (purple) icons represent the placement of the data computer, router, and transceiver. (TIF) [file pone.0090256.s001.tif]

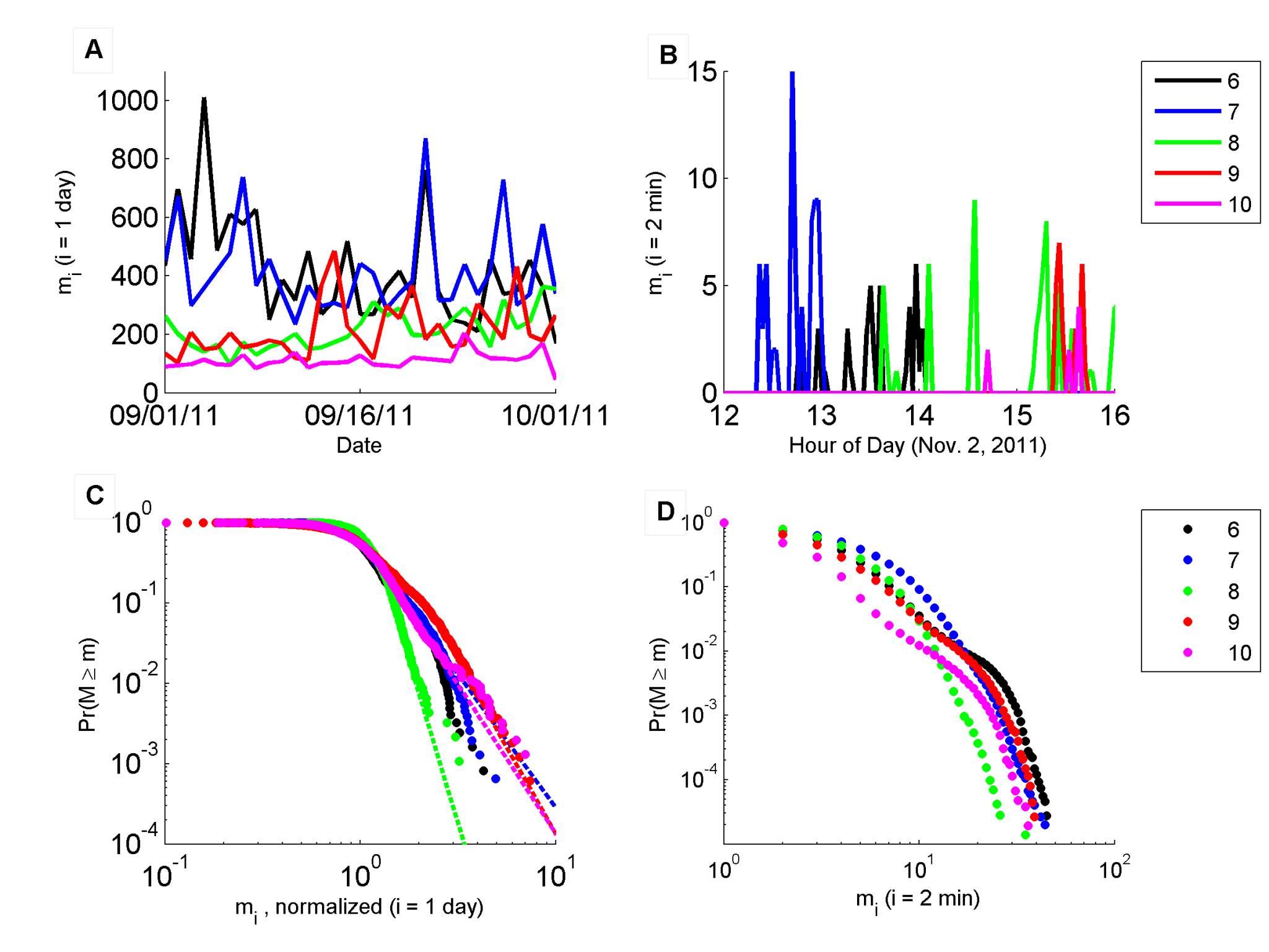

Supplement: Figure S2 — Another snapshot of individual in-home mobility patterns. Time series plots of in-home mobility for participants 6–10 (color coded) for (A) daily-mobility over 31 days starting November 1st of 2011 and (B) 2-minute increments for four hours of November 2, 2011. (C) Day-level data for 5 homes is shown (circles) with best fit power laws (dashed lines), indicating good fit for individual homes but not across homes. Note that as we have plotted the cumulative distribution function for the power laws, the slopes in the plot are −α+1 for each participant's value of α. (D) Data at the two-minute level was not consistent with a power law distribution but is still heavy-tailed. Participant 6's data was not consistent with a power law at the day level (black) with no power law trace in (C). Dates shown were chosen to show one month worth of data for all homes. (TIF) [file pone.0090256.s002.tif]

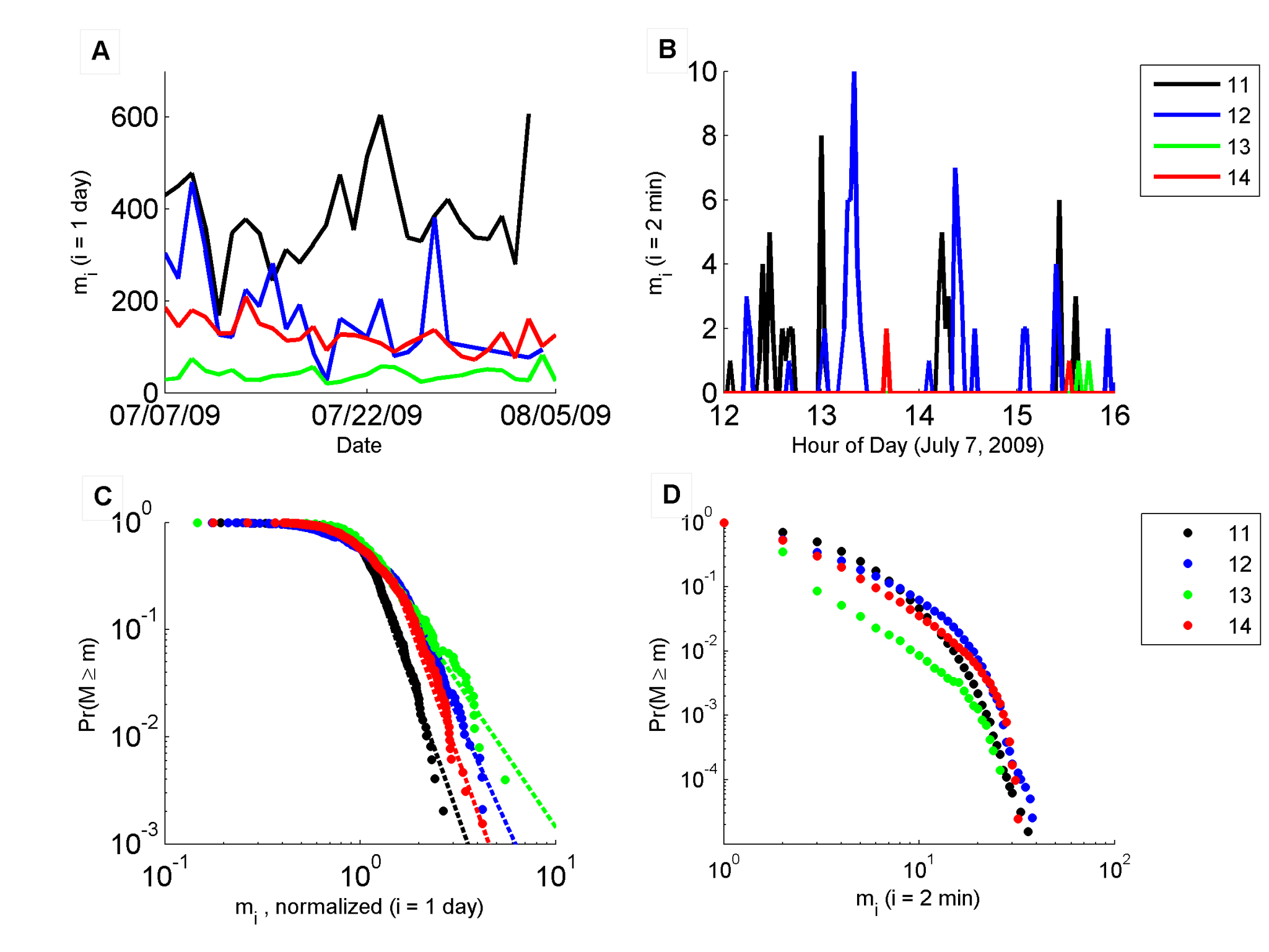

Supplement: Figure S3 — A third snapshot of individual in-home mobility patterns. Time series plots of in-home mobility for participants 11–14 (color coded) for (A) daily-mobility over 31 days starting July 7, 2009 and (B) 2-minute increments for four hours of July 7, 2009. (C) Day-level data for 4 homes is shown (circles) with best fit power laws (dashed lines), indicating good fit for individual homes but not across homes. Note that as we have plotted the cumulative distribution function for the power laws, the slopes in the plot are −α+1 for each participant's value of α. (D) Data at the two-minute level was not consistent with a power law distribution but is still heavy-tailed Dates shown were chosen to show one month worth of data for all homes. (TIF) [file pone.0090256.s003.tif]

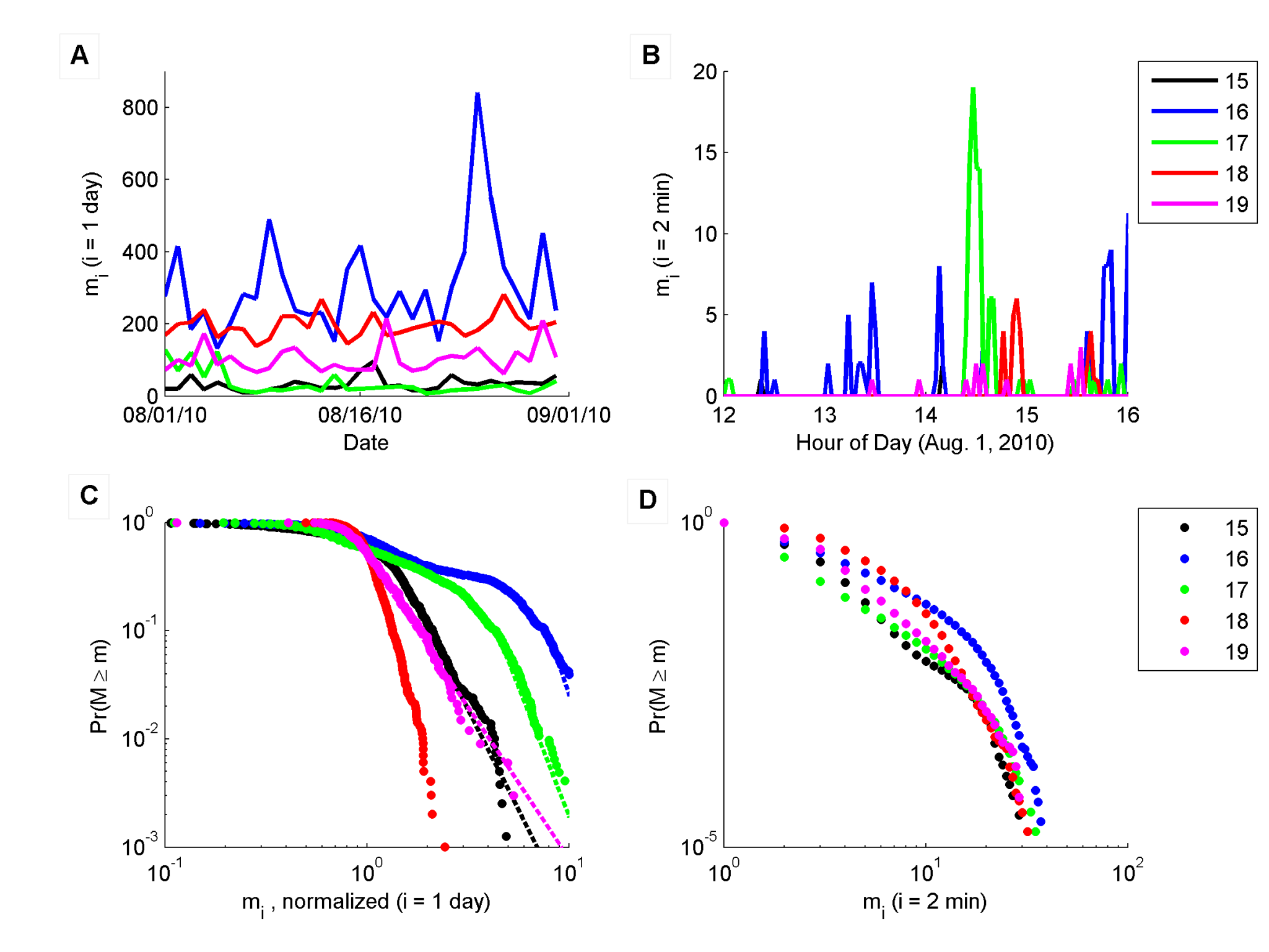

Supplement: Figure S4 — A fourth snapshot of individual in-home mobility patterns. Time series plots of in-home mobility for participants 15–19 (color coded) for (A) daily-mobility over 31 days starting August 1, 2009 and (B) 2-minute increments for August 1, 2009. (C) Day-level data for 5 homes is shown (circles) with best fit power laws (dashed lines), indicating good fit for individual homes but not across homes. Note that as we have plotted the cumulative distribution function for the power laws, the slopes in the plot are −α+1 for each participant's value of α. (D) Data at the two-minute level was not consistent with a power law distribution but is still heavy-tailed Participant 18's data was not consistent with a power law at the day level (red) with no power law trace in (c). Dates shown were chosen to show one month worth of data for all homes. (TIF) [file pone.0090256.s004.tif]
