# Supplementary material for: Regularity and Predictability of Human Mobility in Personal Space
Source: PLoS One. 2014 Feb 27;9(2):e90256. doi: 10.1371/journal.pone.0090256 (PMC3937357; doi:10.1371/journal.pone.0090256)
Supplement: Table S1 — Power law results for mobility in one day increments after normalization for home-specific median mobility and number of sensors installed in the home. The distributional parameters: α, mmin, and mmax are reported along with standard errors αSE and mmin,SE and the p value for the fit (higher p values suggest a better fit). The largest observed value in the data for each home, max(m) is reported for comparison with the largest value for which a power law is consistent (mmax) to quantify the range over which a power law holds. The standard errors combined with the parameter estimates show that even after normalizing for participant and measurement specific effects, there is not a universal power law. Homes 6 and 18 were not consistent with a power law. (DOC) [file pone.0090256.s005.doc]

| Home | *α* | *α_SE_* | *m_min_* | *m_min,SE_* | *p* value | *m_max_* | *max*(*m*) |
| --- | --- | --- | --- | --- | --- | --- | --- |
| 1 | 6.769 | 0.673 | 1.370 | 0.142 | 0.112 | 2.953 | 8.811 |
| 2 | 7.537 | 1.606 | 2.153 | 0.328 | 0.119 | 3.506 | 4.912 |
| 3 | 5.604 | 0.816 | 1.996 | 0.334 | 0.235 | 5.828 | 5.828 |
| 4 | 7.553 | 1.034 | 1.970 | 0.266 | 0.738 | 4.300 | 4.418 |
| 5 | 6.082 | 0.303 | 1.124 | 0.070 | 0.110 | 3.275 | 5.765 |
| 6 | - | - | - | - | - | - | - |
| 7 | 4.319 | 0.173 | 1.082 | 0.069 | 0.119 | 4.745 | 4.745 |
| 8 | 9.042 | 1.054 | 1.265 | 0.071 | 0.859 | 2.901 | 2.901 |
| 9 | 5.319 | 0.571 | 2.423 | 0.413 | 0.761 | 7.052 | 7.052 |
| 10 | 4.699 | 0.242 | 1.197 | 0.096 | 0.197 | 10.644 | 10.644 |
| 11 | 6.191 | 0.689 | 1.128 | 0.086 | 0.535 | 2.528 | 2.528 |
| 12 | 5.018 | 0.526 | 1.528 | 0.124 | 0.478 | 3.866 | 3.866 |
| 13 | 3.684 | 0.303 | 1.051 | 0.102 | 0.302 | 4.821 | 4.821 |
| 14 | 6.058 | 1.224 | 1.556 | 0.193 | 0.178 | 3.788 | 3.788 |
| 15 | 4.713 | 0.382 | 1.658 | 0.192 | 0.472 | 4.493 | 4.493 |
| 16 | 6.546 | 1.644 | 8.256 | 2.143 | 0.951 | 18.064 | 18.064 |
| 17 | 5.772 | 1.049 | 4.481 | 0.754 | 0.549 | 12.407 | 12.407 |
| 18 | - | - | - | - | - | - | - |
| 19 | 3.798 | 0.389 | 0.888 | 0.191 | 0.111 | 5.234 | 5.234 |
