# Supplementary material for: Regularity and Predictability of Human Mobility in Personal Space
Source: PLoS One. 2014 Feb 27;9(2):e90256. doi: 10.1371/journal.pone.0090256 (PMC3937357; doi:10.1371/journal.pone.0090256)
Supplement: Table S2 — Summary of model. Parameters (mean and 95% confidence intervals), significance at the 5% level (denoted by *), interpretation as a percentage change in the expected mobility due to a unit change in the associated independent variable with all other variables held constant, units, and description of variables. (DOC) [file pone.0090256.s006.doc]

| **Parameter** | **Mean** | **95% CI** | | **Significant** | **Percent Change** | **Units** | **Description** |
| --- | --- | --- | --- | --- | --- | --- | --- |
| **Pseudo-R2** | 0.665 | 0.664 | 0.665 | * | - | none | Indication of the quality of the model (model fit) |
| **trantimeM** | -36.442 | -56.385 | -26.512 | * | -100.00% | none | Accounts for missing data |
| **leHour** | -0.076 | -0.322 | 0.173 |  | -7.36% | None | Lagged life event indicator; 1 hour |
| **leDay** | 0.020 | -0.004 | 0.043 |  | 1.98% | None | Lagged life event indicator; 1 day |
| **leWeek** | 0.022 | 0.008 | 0.037 | * | 2.25% | None | Lagged life event indicator; 7 days |
| **leMonth** | 0.032 | 0.019 | 0.044 | * | 3.26% | none | Lagged life event indicator; 28 days |
| **srf_max_temp** | 0.002 | 0.001 | 0.003 | * | 0.21% | Degrees F | Max temperature, 1 day forecast |
| **srf_min_temp** | -0.002 | -0.003 | -0.001 | * | -0.21% | Degrees F | Min temperature, 1 day forecast |
| **srf_prcp** | 0.003 | -0.660 | 0.701 |  | 0.31% | Inches | Precipitation, 1 day forecast |
| **srf_skydesc** | 0.000 | 0.000 | 0.001 |  | 0.04% | none | Qualitative comment |
| **srf_pcpdesc** | 0.000 | 0.000 | 0.000 |  | -0.01% | none | Qualitative comment |
| **srf_tmpdesc** | -0.003 | -0.007 | 0.001 |  | -0.31% | none | Qualitative comment |
| **srf_airdesc** | 0.000 | 0.000 | 0.000 |  | 0.00% | none | Qualitative comment |
| **srf_uvindex** | 0.003 | 0.001 | 0.006 | * | 0.35% | index | The UV index |
| **srf_wndsped** | -0.002 | -0.003 | -0.001 | * | -0.19% | mph | Wind speed |
| **srf_wnddrct** | 0.000 | 0.000 | 0.000 | * | -0.02% | degrees (360) | Wind direction |
| **srf_humidty** | 0.001 | 0.001 | 0.001 | * | 0.11% | percentage | Humidity |
| **srf_dewpoit** | -0.002 | -0.002 | -0.001 | * | -0.19% | none | Dew point |
| **srf_cmflevl** | 0.002 | 0.001 | 0.003 | * | 0.19% | none | Qualitative comment |
| **srf_rain24** | 0.018 | -0.686 | 0.678 |  | 1.78% | inches | Rain forecast for the next day |
| **srf_precpp24** | 0.000 | 0.000 | 0.000 | * | 0.02% | none | Probability of precipitation in the next day |
| **srf_maxd** | -0.001 | -0.001 | 0.000 | * | -0.09% | Degrees F | Max temperature deviation from weekly average |
| **ws** | 0.000 | 0.000 | 0.001 | * | 0.04% | cm/s | Mean walking speed |
| **ws2Min** | 0.000 | 0.000 | 0.001 | * | 0.04% | cm/s | Lagged mean walking speed; 2 minutes ago |
| **wsHour** | 0.000 | 0.000 | 0.001 |  | 0.02% | cm/s | Lagged mean walking speed; 1 hour ago |
| **wsDay** | 0.000 | 0.000 | 0.000 |  | 0.01% | cm/s | Lagged mean walking speed; 1 day ago |
| **wsWeek** | 0.000 | 0.000 | 0.001 |  | 0.02% | cm/s | Lagged mean walking speed; 7 days ago |
| **wsMonth** | 0.001 | 0.000 | 0.001 | * | 0.07% | cm/s | Lagged mean walking speed; 28 days ago |
| **wsM** | 0.014 | -0.001 | 0.030 |  | 1.41% | none | Accounts for missing data |
| **wsM2Min** | 0.055 | 0.036 | 0.078 | * | 5.66% | none | Accounts for missing data |
| **wsMHour** | 0.022 | -0.024 | 0.062 |  | 2.19% | none | Accounts for missing data |
| **wsMDay** | 0.023 | -0.023 | 0.064 |  | 2.34% | none | Accounts for missing data |
| **wsMWeek** | 0.039 | -0.002 | 0.086 |  | 3.98% | none | Accounts for missing data |
| **wsMMonth** | 0.066 | 0.026 | 0.106 | * | 6.86% | none | Accounts for missing data |
| **prws** | 0.003 | 0.003 | 0.003 | * | 0.28% | cm/s | Average of walking speed across the cohort |
| **prwsM** | 0.226 | 0.205 | 0.249 | * | 25.36% | none | Accounts for missing data |
| **minute** | 0.000 | 0.000 | 0.000 |  | 0.00% | none | Minute (0-59) |
| **hr** | -0.001 | -0.001 | 0.000 | * | -0.09% | none | Hour (0-23) |
| **day** | 0.000 | 0.000 | 0.000 |  | 0.01% | none | Day (0-30) |
| **mth** | -0.002 | -0.003 | -0.001 | * | -0.19% | none | Month (0-11) |
| **yr** | -0.022 | -0.025 | -0.020 | * | -2.21% | none | Year (actual; e g., 2011) |
| **numws** | 0.045 | 0.034 | 0.054 | * | 4.60% | count | Number of walks through sensor line |
| **numws2Min** | 0.016 | 0.004 | 0.028 | * | 1.57% | count | Lagged number of walks through sensor line |
| **numwsHour** | -0.007 | -0.033 | 0.017 |  | -0.72% | count | Lagged number of walks through sensor line |
| **numwsDay** | 0.002 | -0.023 | 0.025 |  | 0.16% | count | Lagged number of walks through sensor line |
| **numwsWeek** | 0.003 | -0.027 | 0.026 |  | 0.31% | count | Lagged number of walks through sensor line |
| **numwsMonth** | 0.003 | -0.026 | 0.026 |  | 0.25% | count | Lagged number of walks through sensor line |
| **trantimeM2Min** | -0.153 | -0.159 | -0.147 | * | -14.18% | none | Accounts for missing data |
| **trantimeMHour** | -0.034 | -0.046 | -0.024 | * | -3.38% | none | Accounts for missing data |
| **trantimemMDay** | -0.039 | -0.052 | -0.028 | * | -3.82% | none | Accounts for missing data |
| **trantimeMWeek** | -0.038 | -0.054 | -0.024 | * | -3.76% | none | Accounts for missing data |
| **trantimeMMonth** | -0.042 | -0.055 | -0.027 | * | -4.16% | none | Accounts for missing data |
| **trantime** | -0.004 | -0.004 | -0.004 | * | -0.38% | ms | Mean time to tranistion between rooms |
| **trantime2Min** | -0.002 | -0.003 | -0.002 | * | -0.22% | ms | Lagged mean time to tranistion between rooms |
| **trantimeHour** | 0.000 | -0.001 | 0.001 |  | -0.01% | ms | Lagged mean time to tranistion between rooms |
| **trantimeDay** | 0.000 | -0.001 | 0.000 |  | -0.04% | ms | Lagged mean time to tranistion between rooms |
| **trantimeWeek** | 0.000 | -0.001 | 0.000 |  | -0.04% | ms | Lagged mean time to tranistion between rooms |
| **trantimeMonth** | -0.001 | -0.001 | 0.000 | * | -0.07% | ms | Lagged mean time to tranistion between rooms |
| **numfir** | 0.100 | 0.099 | 0.100 | * | 10.48% | count | Total number of sensor firings |
| **numfir2Min** | -0.006 | -0.006 | -0.005 | * | -0.58% | count | Lagged total number of sensor firings |
| **numfirHour** | 0.001 | 0.000 | 0.002 | * | 0.12% | count | Lagged total number of sensor firings |
| **numfirDay** | -0.001 | -0.002 | 0.000 | * | -0.12% | count | Lagged total number of sensor firings |
| **numfirWeek** | -0.002 | -0.002 | -0.001 | * | -0.16% | count | Lagged total number of sensor firings |
| **numfirMonth** | -0.001 | -0.001 | 0.000 |  | -0.07% | count | Lagged total number of sensor firings |
| **toh** | 0.045 | 0.013 | 0.075 | * | 4.55% | proportion | Time out of house |
| **toh2Min** | 0.007 | -0.014 | 0.026 |  | 0.66% | proportion | Lagged time out of house |
| **tohHour** | 0.021 | 0.013 | 0.029 | * | 2.10% | proportion | Lagged time out of house |
| **tohDay** | 0.020 | 0.014 | 0.026 | * | 2.01% | proportion | Lagged time out of house |
| **tohWeek** | 0.022 | 0.014 | 0.030 | * | 2.21% | proportion | Lagged time out of house |
| **tohMonth** | 0.024 | 0.017 | 0.031 | * | 2.39% | proportion | Lagged time out of house |
| **numactsens** | -0.056 | -0.072 | -0.041 | * | -5.47% | none | Number of active sensors in the home |
| **totnumsens** | 0.032 | 0.016 | 0.046 | * | 3.20% | none | Total number of sensors in the home |
| **fall** | 0.005 | -0.032 | 0.041 |  | 0.50% | none | Indicator from weekly health form |
| **health** | 0.026 | 0.007 | 0.045 | * | 2.59% | none | Indicator from weekly health form |
| **space** | 0.025 | 0.003 | 0.045 | * | 2.57% | none | Indicator from weekly health form |
| **blue** | -0.030 | -0.061 | -0.002 | * | -2.97% | none | Indicator from weekly health form |
| **meds** | 0.028 | 0.012 | 0.046 | * | 2.81% | none | Indicator from weekly health form |
| **hurt** | 0.064 | -0.020 | 0.130 |  | 6.63% | none | Indicator from weekly health form |
| **er** | 0.012 | -0.024 | 0.056 |  | 1.23% | none | Indicator from weekly health form |
| **age** | -0.008 | -0.008 | -0.007 | * | -0.76% | years | Age |
| **sqft** | 0.000 | 0.000 | 0.000 | * | 0.00% | sqft | Square footage of house / apartment |
| **sex** | 0.018 | 0.004 | 0.032 | * | 1.78% | none | Gender |
| **educ** | -0.027 | -0.029 | -0.025 | * | -2.63% | years | Years of education |
| **ses** | 0.012 | 0.012 | 0.013 | * | 1.22% | none | Socioeconomic status |
| **lagMDay** | 0.011 | -0.085 | 0.116 |  | 1.11% | none | Accounts for missing data |
| **lagMWeek** | 0.003 | -0.051 | 0.057 |  | 0.34% | none | Accounts for missing data |
| **lagMMonth** | 0.001 | -0.045 | 0.046 |  | 0.06% | none | Accounts for missing data |
| **formM** | 0.013 | 0.006 | 0.019 | * | 1.26% | none | Accounts for missing data |
| **constant** | 45.515 | 40.501 | 51.424 | * | - | none | Model constant |
| **/lnalpha** | -4.004 | -4.131 | -3.888 | * | - | none | Log of the dispersion parameter |
