# Supplementary material for: Regularity and Predictability of Human Mobility in Personal Space
Source: PLoS One. 2014 Feb 27;9(2):e90256. doi: 10.1371/journal.pone.0090256 (PMC3937357; doi:10.1371/journal.pone.0090256)
Supplement: Table S3 — Proportion of correct mobility estimates with 95% confidence intervals (CI) for different values of largest allowable estimation error (residual size; see Fig. 2A ). (DOC) [file pone.0090256.s007.doc]

| Error (Size of largest Residual) | Proportion of Correct Mobility Estimates | 95% CI | |
| --- | --- | --- | --- |
| 0.1 | 0.91844 | 0.91817 | 0.91870 |
| 0.25 | 0.92573 | 0.92547 | 0.92599 |
| 0.5 | 0.93838 | 0.93813 | 0.93863 |
| 1 | 0.95868 | 0.95845 | 0.95891 |
| 2 | 0.98135 | 0.98116 | 0.98153 |
| 3 | 0.99157 | 0.99142 | 0.99173 |
| 4 | 0.99616 | 0.99603 | 0.99628 |
| 5 | 0.99815 | 0.99804 | 0.99825 |
