# Supplementary material for: Regularity and Predictability of Human Mobility in Personal Space
Source: PLoS One. 2014 Feb 27;9(2):e90256. doi: 10.1371/journal.pone.0090256 (PMC3937357; doi:10.1371/journal.pone.0090256)
Supplement: Table S4 — Estimated, observed, and difference in mobility probabilities according to mobility value (see Fig. 2B ). (DOC) [file pone.0090256.s008.doc]

| m_i_ | P(Observed) | P(Estimated) | Difference |
| --- | --- | --- | --- |
| 0 | 0.913865 | 0.923175 | -0.009311 |
| 1 | 0.028964 | 0.016521 | 0.012443 |
| 2 | 0.018402 | 0.017022 | 0.001380 |
| 3 | 0.012647 | 0.013749 | -0.001102 |
| 4 | 0.008602 | 0.009847 | -0.001245 |
| 5 | 0.004957 | 0.006647 | -0.001691 |
| 6 | 0.003678 | 0.004365 | -0.000688 |
| 7 | 0.002339 | 0.002835 | -0.000496 |
| 8 | 0.001830 | 0.001838 | -0.000008 |
| 9 | 0.001144 | 0.001198 | -0.000055 |
| 10 | 0.000915 | 0.000790 | 0.000124 |
| 11 | 0.000629 | 0.000530 | 0.000099 |
| 12 | 0.000468 | 0.000363 | 0.000106 |
| 13 | 0.000342 | 0.000254 | 0.000088 |
| 14 | 0.000258 | 0.000182 | 0.000076 |
| 15 | 0.000184 | 0.000133 | 0.000051 |
| 16 | 0.000160 | 0.000100 | 0.000060 |
| 17 | 0.000117 | 0.000076 | 0.000040 |
| 18 | 0.000090 | 0.000060 | 0.000031 |
| 19 | 0.000073 | 0.000047 | 0.000026 |
| 20 | 0.000061 | 0.000038 | 0.000023 |
