# Supplementary material for: Regularity and Predictability of Human Mobility in Personal Space
Source: PLoS One. 2014 Feb 27;9(2):e90256. doi: 10.1371/journal.pone.0090256 (PMC3937357; doi:10.1371/journal.pone.0090256)
Supplement: Supporting Information S1 — Additional information on data, methods, and results. (DOC) [file pone.0090256.s009.doc]

## Data and Methods

## Subjects

Data from 19 subjects enrolled in the Intelligent Systems for Assessing Aging Changes (ISAAC) study[1] were used in our analysis. The ISAAC study is a longitudinal community cohort study consisting of standardized clinical and neuropsychological testing combined with a continuous and unobtrusive in-home assessment platform in an elderly population in the Portland, OR (USA) metropolitan area. The study is described in detail elsewhere[1]; here we cover the criteria for study enrollment prior to describing the demographics of the subjects whose data were used in the present study.

Enrollment in the study was based on 5 main criteria: being 80 years of age or older at study enrollment, living without a formal caregiver, living in a residence larger than a one-room studio apartment, being cognitively healthy, and in average health for the age of the participant. There was relaxation of the age requirement for spouses of enrolled participants or minority participants[1]. From this cohort of 265 residents (some cohabitating), we selected a subset of 19 subjects who lived alone and did not have persistent long term technical issues with the in-home assessment platform. Of these 19 subjects, 16 were female and the mean age was 87.4 years (± 6.6 years). This study was approved by the Oregon Health & Science University Institutional Review Board (IRB# 2353).

## In-Home Monitoring Platform

Every home in the ISAAC cohort was equipped with a set of sensors, a computer for personal use, and a computer to collect the sensor data wirelessly through an attached transceiver. The sensor set consisted of two types of sensors – infrared motion sensors and contact sensors. Both types of sensor would transmit a unique code wirelessly when activated, which was detected by the transceiver and time stamped by the attached data computer. The data were uploaded to a remote database each night over the Internet.

One infrared motion sensor was placed in each room of the residence to provide room location information. A set of four motions sensors with a restricted field of view (approximately four degrees) was placed in a linear array in a confined space (such as a hallway) and used to estimate speed of walking[2]. Contact sensors were placed on all entrances/exits in the home and were used to detect when a resident left the home. Figure S1 shows an example floor plan for a residence with the motion sensors, restricted motion sensors, contact sensors, and data computer as well as labels for the associated rooms[1,3,4].

## Analyses

**Power Law Analysis:** The data used in the power law analysis were the mobility, *m_i_*, of all 19 subjects at both *i* = 2 minutes and *i* = 24 hours (calculated from midnight to midnight). *m_i_* , the number of times an individual moves between different rooms in their home, is calculated from the motion sensor data stream as the number of times that sensors firing adjacently in time are different. The motion sensor data stream is constructed as the time ordered sensor stream of all the sensors that fired in the interval, *i*, plus the last sensor firing from the prior interval. From this stream we count the number of adjacent sensors (in the forward-in-time direction only) that are different. For example, if the motion sensor stream for interval *i* was: Living Room->Living Room->Bathroom->Kitchen, then we would set *m_i_* = 2 (for the Living Room->Bathroom and Bathroom ->Kitchen).

To determine whether similar power law behavior (as a data generating process) is consistent for in-home mobility as has been shown to hold for out-of-home mobility[5,6], we followed the guidelines and used the Matlab code by Clauset, et. al[7] to estimate the parameters of and assess the goodness of fit for truncated power laws of the form $p(m)\sim m^{-\alpha}$ for $m_{\min}\leq m$ with a slight refinement for the case of for $m_{\min}\leq m\leq m_{\max}$. The analysis proceeds as follows. First, we assume that a power law holds (as the null hypothesis) and then estimate the parameters, *m_min_*, *m_max_*, and *α*. This is done with a sequential estimation procedure as follows:

1. Estimate *m_min_*, *α*, and the goodness of fit with Kolmogorov-Smirnov (KS) test statistic as described in Clauset, et. al[7].
2. If the p-value from the KS test is greater than or equal to 0.1, stop. *m_max_* is estimated as the largest data point in the set of mobility values.
3. If the p-value from the KS test is less than 0.1, remove all instances of the largest mobility value from the data set and start over at step 1.

This procedure either produces a plausible power law (assessed by the goodness of fit from the KS test, *p*≥0.1) that fits the *largest range* of the mobility data or terminates with no power law found that is plausible with the data (since the mobility data is integer valued, this procedure will terminate in a finite number of steps). This procedure *does not* validate that a power law is the correct data generating mechanism, only that it is a plausible data generating process for the observed data (specifically, we are not testing the goodness of fit against alternative data generating processes). Further, removing data points (step 3) is valid because we are not attempting to model the data outside of the plausible power law range and doing so admits a simple brute force approach to estimate *m_max_*. This procedure was applied for *i* = 2 minutes and 24 hours.

For *i* = 24 hours, we first normalized *m_i_* by the home-specific median over the entire data set for the home and the number of active sensors in the home. This normalization was intended to mitigate the effect on mobility of different home floor plans, sensor placement, number of sensors, and personal levels of mobility. This individual-specific normalization constant was intended to be closely related to radius of gyration[5] used to characterize the size of an individual’s trajectory from cell phone data (out-of-home mobility) and is a function of the time *t* for which the user is observed. The radius of gyration was a crucial parameter that normalized individual mobility patterns in such a way that out-of-home mobility was shown to have a single distribution independent of each individual phone user after the normalization[5]. Since we do not explicitly account for spatial characteristics in our data, we cannot characterize the average size of the trajectory in space but can characterize the average size (count) of the trajectory in time (i.e, the average amount of mobility observed as a function of time). We selected the median day level mobility as this normalization constant for several reasons. First, the median is a more robust measure of central tendency than the mean. Second, for distributions that are non-negative and non-symmetric, the sample median is not leveraged by large observations as is the sample mean. Third, the median is a value taken by the observed process (it is an integer whereas the mean, for example, may not be). Finally, we also divided the daily mobility by the number of active sensors in each home on each day to account for the “opportunity space” in which we have to observe transitions (e.g., number of different rooms) and to adjust for technical issues with sensors (e.g., a broken sensor that does not capture mobility) both of which may impact longitudinal and cross sectional comparisons if not accounted for. We further believe that the median helps normalize the effect of an individual’s mobility for cross-sectional comparison of scaling exponents because it tends to contain some of the same distance information that is unmeasured but implicitly present in the count mobility time series under analysis. We acknowledge that while this appears to be the most appropriate normalization for these data, it may be that we have not captured the same essence in these data that has been captured in the cell phone data by the radius of gyration. As a result, the the lack of “universality” we report in the 17/19 homes for which a power law is a plausible fit at the day level may be a result of our choice of normalization. However, no constant normalization would cause the 2/19 homes at the day level or all 19 homes at the 2 minute level to follow a power law. Therefore - and perhaps most importantly - we argue that a power law distribution is insufficient to adequately describe these data, thus we did not find similar results as with other studies on mobility outside of the home. No normalization was applied for *i* = 2 minutes as there was a large number of zeros in the data (*m_i_*=0 for approximately 91.8% of the data at 2 minute intervals) making mean or median mobility less useful or interpretable, and the range of the data at 2-minute intervals is not largely different between homes at this granularity. Figs. S2-S4 shows the results for the 14 participants not shown in the main paper while Table S1 shows the power law parameter estimates for all participants.

Some further comments on the power law analysis and the relationship between our work and other studies on out of home mobility are in order. Because of the importance of the work, we attempted to adapt much of the analysis by Gonzalez et.al.[5], regarding scaling exponents and universality in out-of-home mobility to analyze data collected in-the-home. While we found that these same results do not appear to hold in the home, this should not cast doubt on the work of Gonzalez or others. Rather it suggests that in-home mobility may be a fundamentally different phenomenon than out-of-home mobility. While future work will be required to delineate and characterize all the differences and similarities between these two aspects of human mobility, there are several reasons to believe that in-home and out-of-home mobility may be fundamentally different: people customize their homes (which rooms are used for what, furniture placement, etc…), the size of the home is bounded in a way that is substantially different than the “outside world”, people routinely visit their entire home space, people cannot explore “new” home space as they can with the outside world (e.g., traveling further than before or to new locations), and our study participants were followed for up to five years (3 years, on average) instead of only 6 months, giving ample time for behavioral change, seasonal effects, and other phenomena that may not be observed in shorter studies. However, similar to the results reported by Gonzalez (and others) we were able to find substantial regularity and predictability in the observed mobility patterns. This suggests that much like the structure found in out-of-home mobility, significant structure also exists in the way in which humans move about their homes.

**Contextual Model – Variables:** The data used in this study can be loosely broken up into 7 categories. Here we describe the origin of the data from the following categories: behavioral, weather, self-report, peer-reference, time dependence, missing data, and physical environment. All data were sampled at 2 minute intervals over the course of the study. Table S2 contains units and a brief description of all independent variables. Note that all variables here are reported in the units in which they were measured (thus remaining consistent with measurements stored in the databases from which the data were gathered); non-SI units have been converted to SI units in the paper only.

*Behavioral* – The dependent variable as well as several of the independent variables are categorized as behavioral. This categorization is based on the fact that all these variables are measures of movement, performance or patterns of these over time. Mobility, *m_i_*, is calculated from sensor data as the number of times two adjacent sensors fire in different rooms ([8], and as described above). The total number of sensor firings (numfir) is a count variable calculated as the total number of times any sensor fired in the interval. Walking speed (ws) is calculated as the average over all estimated walking speeds (from a walking speed senor line [2]) during each interval. The number of walks (numws) is a count variable recording the number of times a walking speed was estimated [9]. The transition time (trantime) is the average time to move between rooms and is calculated over all recorded transitions in the interval. The time out of house (toh) is calculated based on using door sensors to detect when residents left the home and using motion sensors to ensure that no one is in the home.

*Weather* – Weather data was collected from initialization data for the National Weather Service’s Global Forecast System (GFS) [10] with commercial reanalysis provided by Custom Weather, Inc. [11]. Sixteen independent variables describing weather and climate were used in this study and geocoded to the participant data via zip code. All weather variables were same-day forecasts. Temperature variables, measured in degrees Fahreinheit (and converted to Celsius for the figures in the paper), included the maximum temperature (srf_max_temp), minimum temperature (srf_min_temp), maximum deviation from the weekly average temperature (srf_maxd), and a qualitative descriptor (srf_tmpdesc). Precipitation was represented by the probability of precipitation in the next 24 hours (srf_prcpp24), the total amount of precipitation (srf_prcp), the amount of rain (srf_rain24), both in inches, and a qualitative description (srf_pcpdesc). Wind was represented by speed (srf_wndsped) in miles and direction (srf_wnddrct) in degrees. Dewpoint (srf_dewpoit), humidity (srf_humidity), and ultraviolet index (srf_uvindex) were also included. The remaining weather variables consisted of qualitative variables describing sky conditions (srf_skydesc), perceived comfort level (srf_cmfdesc), and air conditions (srf_airdesc).

*Self-Report* – The majority of self-report variables were gathered by asking participants to respond to a weekly health survey consisting of 9 questions (this has since been expanded to 12, but the additional questions are not used here as they are not available for the entire monitoring time). This survey was administered online and was made available every Monday morning. Participants were not required to fill out the survey, and the survey remained available until it was filled out. A follow up call was made if the form was not completed within a week of becoming available. 5.1% of the forms were not completed for which participants received a call. All variables were coded as 0 or 1 indicating a yes or no answer to whether the subject experienced a fall in the last week (fall), felt as though their health had limited their ability in the last week (health), had made a change to their living space such as moving furniture in the last week (space), felt downhearted or “blue” for at least three days in the last week (blue), had a medication change in the last week (meds), had any non-falling injuries in the last week (hurt), or had a trip to the emergency room or hospital in the last week (er). Two additional questions from the form were combined into one “life event” category (le) indicating whether the subject had been away from home overnight or had visitors who stayed in the home for which the dates of incidence were used. Four additional self-report variables were recorded at baseline: socio-economic status (ses)[12], years of education (educ), gender (sex), and age in years (age; we collected these data once but used their true age at each sampled point).

*Peer-Reference* – One variable was used as a peer reference variable – the average walking speed of the peer group (prws; in this case, the peer group was defined as the remaining 18 participants who are all of similar age) calculated as an average walking speed of all subjects for whom we estimated a walking speed in the interval.

*Time Dependence* – Several variables were used capture time dependence, seasonality, and cyclical patterns (e.g., circadian, infradian, and ultradian rhythms). Minute, hour (hr), day, month (mth), and year (yr) were included as count variables to track changes on different time scales. We lagged all behavioral variables at 2 minutes (one sample), one hour, one day, one week, and one month (28 days). Lagged variables are named with the lag appended to the regular name (2Min, Hour, Day, Week, or Month; ex., walking speed for a one week lag is “wsWeek”; see Table S2). Additionally, the life events indicator (le) was lagged at one hour, one day, a week, and a month to capture the influence of visitor and travel patterns on mobility.

*Missing Data* – To account for missing data, we used dummy variables [13]. We included variables to account for missing walking speeds and all missing lagged walking speeds, missing peer reference walking speed, transition time and all lagged transition times, missing entries to the health form, and missing lags (this accounts for not observing variables prior to the start of our study, thus not being able to have lagged variables associated with the earliest observations). Missing variables are named with an ”M” appended after the variable name but before any appended lag value (ex., a variable accounting for missing walking speed lagged by one week is wsMWeek; see Table S2).

*Physical Environment* – The final category accounts for heterogeneity between participants’ home space and the measurement (sensor) system. We included a variable accounting for the size of participants’ homes (sqft), the number of sensors deployed in the home (totnumsens), and the number of working sensors (numactsens). This last variable accounted for data loss during the time between when a sensor would stop working (e.g., due to a dead battery) and when a technical research assistant could get into the home space and fix the problem. A constant was also included in the model.

**Contextual Model – Statistical Analysis:** Our approach for including context into a model for mobility involved regressing mobility onto the 88 independent variables outlined above. 88 independent variables is perhaps an order of magnitude larger than the number of variables often used in applied regression models and may raise concerns over whether the model may overfit the data. However, we argue that our approach is not at risk for overfitting for several reasons. First and most importantly, because of the amount of time in which we have been monitoring our study participants our data set contains almost 15 million samples with which to estimate the 88 model parameters. This is a ratio of approximately 170,000 samples to each independent variable included, much larger than many other studies (many other study designs do not even gather 170,000 samples). Further, all but five of our variables vary (on different time scales) over the course of the monitoring period (age, square footage of the residence, sex, education, and socioeconomic status do not in this cohort), so even though the number of subjects included in our study is smaller than that used for many purely cross-sectional analyses, we have few variables that are purely cross-sectional in nature (we agree it would be incorrect to put, for example, 88 time-invariant variables in a model with only 19 subjects no matter the amount of longitudinal data). Finally, the results on model fit and predictability use only 2 million samples to fit the model (a ratio of approximately 22,700 data points for each independent variable included) and test on the entire data set, thus approximately 86% of the data used to evaluate our main conclusions are not used to estimate the model parameters. Given these arguments we feel justified in including a seemingly large number of independent variables in the contextual model.

As mobility is a non-negative integer-valued variable, we used a count regression model. The most common count regression models include the Poisson regression model (prm) and the negative binomial regression model (nbrm), where the nbrm can be viewed as an extension of the prm that can handle overdispersion by including a parameter for unobserved heterogeneity[14] (α; see Eq. 1 in the paper). In addition to fitting these models, we also fit zero-inflated versions of both of these models to determine whether there may have been more observed zeros than can be accounted for with non-zero-inflated models. We found that the nbrm model offered a substantially better fit than the prm (the nbrm had a Bayesian Information Criterion 974.36 lower than the prm) and there was significant evidence of overdispersion (Likelihood-ratio test of no overdispersion: *G^2^* = 988.87, *p*< 0.0001). We were unable to get the zero-inflated prm or zero-inflated nbrm to converge to a solution, thus neither model was considered a reasonable candidate for the data generating process underlying human mobility.

After identifying the nbrm as the best model for the data out of those considered, we fit the model in Stata (StataCorp. 2011. *Stata Statistical Software: Release 12*. College Station, TX: StataCorp LP) using command nbreg. After fitting and verifying the model was significant (χ^2^(87)> 1.24e+06; *p*<0.0001), the nbrm was subsequently fit 100 times with 2,000,000 randomly drawn samples (at each iteration) to estimate 95% confidence intervals on the parameter estimates which are robust to autocorrelation and heteroskedasticity (we note that *m_i_* and trantime may be endogenous; we did not attempt to account for this[15]). The results are shown in Table S2 along with McFadden’s pseudo R2 [14], a measure of fit for non-linear regression models, and the log of the overdispersion parameter, α (Eq. 1 in the paper). Of the 88 variables used in the model, 57 were found to be significantly associated with mobility as shown in Table S1. Table S1 also shows the percent change in expected mobility due to a unit change in the associated variable, holding all other variables at fixed values.

After identifying the model, we characterized the predictability, defined as prediction accuracy, in two ways as described in the paper (Fig. 2). First, we estimated the mobility, $\hat{m}_{i}$, for all 14,920,560 samples after fitting the model with 2,000,000 samples. We then calculated the prediction error (residuals), $m_{i}-\hat{m}_{i}$ , and the proportion of estimates whose prediction error was smaller than a certain value for several levels of prediction error. This is shown in Table S3 (see also Fig. 2A in the paper). We then calculated the observed and predicted mobility counts across the whole data set (using the same model) for values of mobility between 1 and 20. This accounts for over 99.97% of the observed mobility, and is displayed in Table S4 (see also Fig. 2B in the paper). To calculate the probability density functions and probability of no transitions (as in Fig. 3 and 4), Stata command prgen from the SPost package[14] was used with following variables set to specific values: trantimeM=0 , leHour=0, leDay=0, leWeek=0, leMonth=0, ws=60 cm/s, ws2min=60 cm/s wsHour=60 cm/s, wsDay=60 cm/s, wsWeek=60 cm/s, wsMonth=60 cm/s, prws=80 cm/s, numws=1, numws2Min=1, numwsHour=1, numwsDay=1, numwsWeek=1, numwsMonth=1, trantime=2 seconds, trantime2Min=2 seconds, trantimeHour=2 seconds, trantimeDay=2 seconds, trantimeWeek=2 seconds, trantimeMonth=2 seconds, numfir=1, numfir2Min=1, numfirHour=1, numfirDay=1, numfirWeek=1, numfirMonth=1, toh=0, toh2Min=0, tohHour=0, tohDay=0, tohWeek=0, tohMonth=0, fall=0, health=0, space=0, blue=0, meds=0, hurt=0, er=0, sex=female, all missing data flags set to 0 (no missing data), and the remaining variables set to their mean values. This set of data represents a healthy female elderly woman of average age (with respect to the ISAAC cohort), no reported health issues, no missing data or recent overnight visitors/travel, nominal behavioral parameters, and who is walking 25% slower than her peers at present. Thus, the data represented in Fig. 3 and Extended Data Fig. 5 represents an expected mobility profile for such a woman. Similar mobility profiles for other people can be queried from the proposed model in the same way.

In order to determine regularity as described in the main paper, we looked at the effect of time-dependence on mobility both directly as the effect of the time variables included in the model (see also Table S2; minute, hr, day, mth, and yr) and the lagged variables (multiple lags – 2-minute, hour, day, week, and month - for each of the following variables: ws, numws, trantime, numfir, and toh). This allows for interpretation as both the direct effect of time and the indirect effects of time with the patterns of the other behavioral variables on specific cycles. The lagged variables also allow determination of whether there are correlations at multiple time scales. This is because if a variable is statistically significant in the model - as are all the variables ws, numws, trantime, numfir, and toh (see Table S2) – then it affects mobility. If the lag is also significant, then the same variable (just shifted in time) also affects mobility at a different point in time, thus the mobility values themselves are correlated through the original variable (at least). This indirect approach is used over something simpler (such as the autocorrelation or another higher order statistic) because the mobility process is non-stationary. Both the mean (rate) and variance of mobility change from sample to sample and thus something such as the autocorrelation does not have the simple form it would if the mobility process were stationary (e.g., R(s,t)≠R(s+ᵼ,t+ ᵼ)), and is thus less interpretable and may not be calculable.

Given this approach to quantify regularity, we found the following: time was statistically significant at the hour, month, and year level suggesting both infradian and ultradian mobility cycles. We did not find the day variable significant, but this may be because we coded day as day of month, not day of week (which we plan to do in future work). The lagged variables were significant as follows (see also Table S2): ws was significant at a 2 minute lag and a one month lag, numws was significant at a 2 minute lag, trantime was significant at lags of 2 minutes and one month, numfir was significant at 2 minutes, an hour, a day, and a week, and toh was significant at lags of one hour, one day, one week, and one month. The analysis of the lagged variables strengthen the evidence for the presence of infradian and ultradian mobility cycles, while also suggesting the presence of circadian cycles and that correlations in mobility patterns exist at multiple time scales (at least at 2 minutes, an hour, a day, and a week). Taken together, this gives strong evidence for a high level of regularity in human mobility patterns in-the-home.

# References

1. Kaye JA, Maxwell SA, Mattek N, Hayes TL, Dodge H, et al. (2011) Intelligent Systems For Assessing Aging Changes: home-based, unobtrusive, and continuous assessment of aging. J Gerontol B Psychol Sci Soc Sci 66 Suppl 1: i180-190.

2. Hagler S, Austin D, Hayes TL, Kaye J, Pavel M (2010) Unobtrusive and ubiquitous in-home monitoring: a methodology for continuous assessment of gait velocity in elders. IEEE Trans Biomed Eng 57: 813-820.

3. Hayes T, Pavel M, Adami A, Larimer N, Tsay I, et al. (2007) Distributed Healthcare: Simultaneous Assessment of Multiple Individuals. Pervasive Computing 6: 36-43.

4. Hayes TL, Abendroth F, Adami A, Pavel M, Zitzelberger TA, et al. (2008) Unobtrusive assessment of activity patterns associated with mild cognitive impairment. Alzheimers Dement 4: 395-405.

5. Gonzalez MC, Hidalgo CA, Barabasi AL (2008) Understanding individual human mobility patterns. Nature 453: 779-782.

6. Song C, Koren T, Wang P, Barabasi AL (2010) Modelling the scaling properties of human mobility. Nature Physics 6: 818-823.

7. Clauset A, Shalizi CR, Newman MEJ (2009) Power-law distributions in empirical data. SIAM Review 51: 661-703.

8. Campbell IH, Austin D, Hayes TL, Pavel M, Riley T, et al. Measuring changes in activity patterns during a norovirus epidemic at a retirement community; 2011. pp. 6793-6796.

9. Kaye J, Mattek N, Dodge H, Buracchio T, Austin D, et al. (2012) One walk a year to 1000 within a year: continuous in-home unobtrusive gait assessment of older adults. Gait Posture 35: 197-202.

10. National Weather Service (1979-2013) Global Forecast System: , National Oceanic and Atmospheric Administration, U.S. Department of Commerce, Washington D.C.

11. Custom Weather Inc. (2006-2013) Two Week Extended Forecasts , San Francisco, CA.

12. Hollingshead A (1975) Four Factor Index of Social Status. Yale University.

13. Allison P (2002) Missing Data: Sage.

14. Long J, Freese J (2006) Regression models for categorical dependent variables using Stata: Stata Corp LP. 527 p.

15. Cameron A, Trivedi P (1998) Regression Analysis of Count Data: Cambridge University Press. 566 p.
